# Vertically Aligned Silicon Carbide Nanowires/Boron Nitride Cellulose Aerogel Networks Enhanced Thermal Conductivity and Electromagnetic Absorbing of Epoxy Composites

**DOI:** 10.1007/s40820-022-00863-z

**Published:** 2022-04-30

**Authors:** Duo Pan, Gui Yang, Hala M. Abo-Dief, Jingwen Dong, Fengmei Su, Chuntai Liu, Yifan Li, Ben Bin Xu, Vignesh Murugadoss, Nithesh Naik, Salah M. El-Bahy, Zeinhom M. El-Bahy, Minan Huang, Zhanhu Guo

**Affiliations:** 1 Key Laboratory of Materials Processing and Mold (Zhengzhou University), Ministry of Education; National Engineering Research Center for Advanced Polymer Processing Technology, Zhengzhou University, Zhengzhou, 450002 People’s Republic of China; 2 Department of Chemistry, College of Science, Taif University, P. O. Box 11099, Taif, 21944 Saudi Arabia; 3 Mechanical and Construction Engineering, Faculty of Engineering and Environment, Northumbria University, Newcastle upon Tyne, NE1 8ST UK; 4 Integrated Composites Laboratory (ICL), Department of Chemical and Biomolecular Engineering, University of Tennessee, Knoxville, TN 37996 USA; 5 Advanced Materials Division, Engineered Multifunctional Composites (EMC) Nanotech LLC, Knoxville, TN 37934 USA; 6 College of Materials Science and Engineering, Taiyuan University of Science and Technology, Taiyuan, 030024 People’s Republic of China; 7 Department of Mechanical and Manufacturing Engineering, Manipal Institute of Technology, Manipal Academy of Higher Education, Manipal, Karnataka 576104 India; 8 Department of Chemistry, Turabah University College, Taif University, P. O. Box 11099, Taif, 21944 Saudi Arabia; 9 Department of Chemistry, Al-Azhar University, Nasr City, Cairo, 11884 Egypt

**Keywords:** Epoxy, Ice template, Vertical alignment, Thermal conductivity, Multifunctionality

## Abstract

With the innovation of microelectronics technology, the heat dissipation problem inside the device will face a severe test. In this work, cellulose aerogel (CA) with highly enhanced thermal conductivity (TC) in vertical planes was successfully obtained by constructing a vertically aligned silicon carbide nanowires (SiC NWs)/boron nitride (BN) network via the ice template-assisted strategy. The unique network structure of SiC NWs connected to BN ensures that the TC of the composite in the vertical direction reaches 2.21 W m^−1^ K^−1^ at a low hybrid filler loading of 16.69 wt%, which was increased by 890% compared to pure epoxy (EP). In addition, relying on unique porous network structure of CA, EP-based composite also showed higher TC than other comparative samples in the horizontal direction. Meanwhile, the composite exhibits good electrically insulating with a volume electrical resistivity about 2.35 × 10^11^ Ω cm and displays excellent electromagnetic wave absorption performance with a minimum reflection loss of − 21.5 dB and a wide effective absorption bandwidth (< − 10 dB) from 8.8 to 11.6 GHz. Therefore, this work provides a new strategy for manufacturing polymer-based composites with excellent multifunctional performances in microelectronic packaging applications.

**Supplementary Information:**

The online version contains supplementary material available at 10.1007/s40820-022-00863-z.

## Highlights

- Cellulose aerogel with vertically oriented structure was obtained by constructing a vertically aligned SiC nanowires/BN network via the ice template assisted strategy.
- The thermal conductivity of the composite in the vertical direction reaches 2.21 W m^−1^ K^−1^ at a low hybrid filler loading of 16.69 wt%, which was increased 890% compared to pure epoxy.
- The composite exhibits good electrically insulating with a volume electrical resistivity about 2.35×10^11^ Ω cm, and displays excellent electromagnetic wave absorption performance.

**Supplementary Information:**

The online version contains supplementary material available at 10.1007/s40820-022-00863-z.

## Graphical abstract

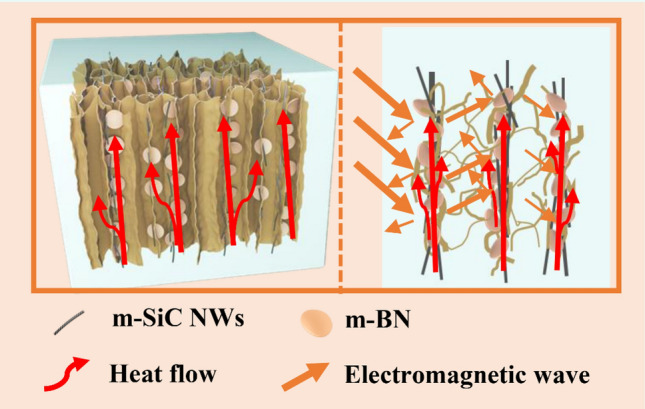

## Introduction

With the innovation of the third-generation semiconductor technology, electronic equipment has shown a development trend of multi-function, miniaturization and integration [1–4]. As a result, a large amount of heat generated inside the equipment continue to accumulate, which seriously affects its reliability and service life [5]. Polymer materials have been widely used in the sealing and interface bonding of various electronic devices due to their excellent mechanical properties, good insulation and unique chemical stability [6–8]. However, the thermal conductivity (TC) of polymer materials is generally low (usually no more than 0.5 W m^−1^ K^−1^), which limits their wide-ranging applications in the microelectronic packaging industry [9–11]. How to improve the TC of polymer materials and make them better apply to the field of thermal management has become an important problem that needs to be solved urgently [12].

Compared with the difficulty to change the molecular chain structure of the intrinsic polymer to improve TC, it is simple and practical to add a filler with high TC into the polymer [13–15]. Generally, such thermally conductive fillers mainly include metal oxides (e.g., Al_2_O_3_, ZnO and MgO) [16], metal particles (e.g., Cu, Zn and Ag) [17], aluminum nitride (AlN) [18], boron nitride (BN) [19], silicon carbide (SiC) [20], graphene [21] and carbon nanotubes [22], etc. Among them, BN is considered to be the most promising two-dimensional material because of its low density, excellent electrical insulation, oxidation resistance and chemical stability properties [23, 24]. Unfortunately, a large amount of BN are often needed in the composite to achieve a satisfactory heat dissipation effect due to the existence of interface thermal resistance between polymer matrix and fillers, which is bound to sacrifice the excellent toughness of the matrix and the mechanical properties of the composite [25, 26]. Therefore, it is of great significance to use less BN fillers to obtain composites with a better TC in high power density electronic devices.

In recent years, compared with simple blending, two strategies of driving the horizontally orientation of BN [27] and building a three-dimensional (3D) BN thermally conductive network [28] can effectively construct heat conduction paths to improve the TC at a low BN content. In the former orientation applications, composites mostly appear in the form of films. For example, Yang et al. [29] fabricated polyvinyl alcohol/boron nitride composite film with high in-plane TC (19.99 W m^−1^ K^−1^) via the combination of electrostatic spinning and hot-pressing technique. Wu et al. [30] reported a BN nanosheet/polymer composite film with superior in-plane TC of around 200 W m^−1^ K^−1^ and extremely low through-plane TC of 1.0 W m^−1^ K^−1^. Although these film-like composites have considerable in-plane TC, the heat dissipation of microelectronic devices is mainly through the rapid transfer of accumulated heat energy from the heat source to the heat sink in a short vertical direction [31, 32]. The TC of the film-like composites in the vertical direction is quite low, which hinders their large-scale use in actual production. The latter uses fillers to establish a spatially interconnected thermally conductive network structure to improve the overall TC of the composite [33]. Chen et al. [34] prepared BN-polyvinylidene difluoride (PVDF) 3D scaffold by removing the sodium chloride salt template method and found that the TC of the final epoxy/BN-PVDF was 1.227 W m^−1^ K^−1^. Zhou et al. [35] synthesized a 3D interconnective cross-linking polystyrene (c-PS)/BN composite foam with a TC of 1.28 W m^−1^ K^−1^ and found that the composite foams exhibited low density and dielectric constants. Although the 3D heat conduction paths constructed by BN in the matrix improves the overall TC of the bulk composite to a certain extent, the TC in the vertical direction cannot be effectively improved by relying on BN alone [36].

Therefore, on one hand, one-dimensional materials (e.g., carbon nanotubes, silver nanowire and SiC nanowire) as a thermally conductive bridge to connect BN were introduced [37], and on the other hand, various strategies (e.g., suction filtration method, magnetic field method and electric field method) were adopted to achieve vertical alignment of fillers [38–40]. One-dimensional SiC nanowire overcomes the defects of traditional SiC materials and has been widely used in aerospace, chemical, electronics and other industrial fields due to its excellent high-temperature strength, good thermally conductive performance, high wear resistance and corrosion resistance [41]. Xiao et al. [31] successfully prepared epoxy-based composites with excellent through-plane TC (4.22 W m^−1^ K^−1^) by constructing a free-standing and vertically aligned SiC nanowires/BN framework through modified filtration strategy. Kim et al. [42] fabricated a directional thermally conductive BN-Fe_3_O_4_/SiC binary filler epoxy composite by introducing Fe_3_O_4_ particles on the BN surface via magnetic alignment technology. The obtained composite not only has excellent thermal management performance, but also has a high storage modulus. Unfortunately, these methods have high technical requirements, and many factors such as density mismatch, electric field distribution, interaction between magnetic ions and uncontrollable grafting sites need to be considered [43].

In addition, with the rapid development of the 5G era, microelectronic devices bring us convenience while carrying potentially serious electromagnetic pollution [44–46]. Efficient microwave absorbing materials have attracted a lot of attention in recent years; however, few studies have combined excellent thermal management and microwave absorption properties simultaneously [47–50]. In this work, based on the previous research work [51, 52] about the cellulose aerogel obtained by the ice template method, the unique structure with a small amount of SiC nanowires (SiC NWs) vertically connected to BN was obtained by modifying the fillers and combining with directional freezing technology. The finally obtained epoxy composite not only exhibits excellent thermal management capability in the vertical direction, but also displays excellent electromagnetic wave absorption performance, which is attributed to the good synergistic effects of SiC NWs and BN in both function and structure.

## Experimental

### Materials

Hexagonal boron nitride powder (*h*-BN, ~ 10 µm, 99.9%) was purchased from Hefei AVIC Nano Technology Development Co., Ltd., China. SiC nanowires (SiC NWs, diameters: 0.1–0.5 μm, length: 20–50 μm) were supplied by Xuzhou Hongwu Nano Material Co., Ltd., China. Sodium hydroxide (NaOH, AR, 96%), Urea (H_2_NCONH_2_, AR, 99%) and cellulose with a length of ≤ 25 mm were obtained from Shanghai Aladdin Reagent Co., Ltd., China. Boric acid (H_3_BO_3_, AR, ≥ 99.5%) and epichlorohydrin (ECH, C_3_H_5_ClO, 1.183 g cm^−3^) were supplied by Shanghai McLean Biochemical Technology Co., Ltd., China. Epoxy (EP, E-44) and amine curing agent 593 were provided by Evergreen Chemicals Technology Co., Ltd., China. All reagents were of analytical grade and used without any further purification.

### Preparation of Cellulose/m-SiC NWs/m-BN Aerogel (CA/m-SiC/m-BN)

Before synthesizing aerogels, the surface of *h*-BN and SiC NWs needs to be modified for better connection and dispersion. Specifically, the original *h*-BN was exfoliation and modified by putting BN and boric acid (BA) in a planetary ball mill at a mass ratio of m(BN)/m(BA) = 1:10 for 36 h at 400 rpm and then subjected to a series of operations of centrifugation, washing and drying to obtain the modified BN, marked as m-BN. For the modification of SiC NWs (m-SiC NWs), commercial SiC NWs were oxidized at a high temperature of 1300 °C with an air atmosphere in a tube furnace for 5 min.

The cellulose/m-SiC NWs/m-BN aerogel was obtained by the corresponding hydrogel through directional freezing followed with freeze drying steps. Firstly, the alkaline solution was prepared by mixing NaOH/urea/deionized water at a weight ratio of 7:14:79. Then, 5 g cellulose was added into 50 mL above solution and stirred evenly and put it in a refrigerator at − 10 °C for 1 h. After that, different masses of m-BN (0.5, 1.0, 1.5 and 1.8 g) followed with a small amount of m-SiC NWs were successively added and magnetically stirred for 2 h to obtain a cellulose/m-SiC NWs/m-BN solution. Next, 10 mL cross-linker ECH is introduced and the obtained mixed paste solution was poured into a mold with an internal diameter of 20 mm and a Cu disk substrate, and cross-linked for regeneration at 60 °C for 5 h in an oven. After that, the formed cellulose/m-SiC NWs/m-BN hydrogels were soaked in deionized water to remove the residual reactants (NaOH, urea and ECH) and followed by directional freezing treatment with liquid nitrogen in the mold for 10 min. Finally, the frozen cellulose/m-SiC NWs/m-BN hydrogels were then transferred to a freeze drier at − 80 °C for 72 h to get cellulose/m-SiC NWs/m-BN aerogels. To simplify writing, cellulose/m-SiC NWs/m-BN aerogel was marked as CA/m-SiC/m-BN.

### Preparation of CA/m-SiC/m-BN/EP Composites

The CA/m-SiC/m-BN/EP composites were fabricated via a vacuum-assisted impregnation method. In brief, 10 g EP and 2 g curing agent 593 were magnetically stirred in a water bath (80 °C) to obtain a homogeneous solution. The CA/m-SiC/m-BN aerogels were immersed into above solution in a vacuum system and cured in an oven at 60 °C for 3 h and 80 °C for 2 h. Eventually, the composites denoted as CA/xm-SiC/ym-BN/EP (x and y correspond to the mass (g) of m-SiC and m-BN) were obtained. In addition, CA/m-SiC/EP and CA/m-BN/EP with the same synthesis method, and CA/m-SiC/m-BN/EP composites by a simple blending (CA/m-SiC/m-BN/EP_blend_) were also prepared for comparison.

### Characterization

The scanning electron microscopy (SEM, JSM-6380, Japan) was applied to observe the morphologies of m-BN, m-SiC NWs and the microstructure of the CA/m-SiC/m-BN network. Atomic force microscopy was conducted (AFM, Bruker MultiMode 8) to investigate the lateral size and thickness of m-BN. Fourier transform infrared spectroscopy (FT-IR) was tested in the range of 500–4000 cm^−1^ using Nicolet NEXUS 870 spectrometer. Elemental scanning was achieved by X-ray photoelectron spectroscopy (XPS, ESCALAB 250 Xi). The filler loading of different samples was estimated by a thermal gravimetric analyzer (TGA, 209 F3, Netzsch). Contact angle was obtained from contact angle measurement (CD-100D, innuo-instruments, Shanghai). Volume resistivity was measured by an Electrometer (Tektronix, 6517B, America) at room temperature. The *K* was measured by a Hot Disk Thermal Constant Analyzer (Hot Disk TPS 2500S, Sweden), and an infrared thermal camera (E60, FLIR) was employed to measure the change of sample surface temperature over different heating and cooling time. A vector network analyzer (VNA, Keysight N5222B, USA) was used to measure the electromagnetic parameters of different samples with the coaxial method in the frequency range of 2–18 GHz.

## Results and Discussions

### Characterization of m-BN, m-SiC NWs

**Fig. 1:**
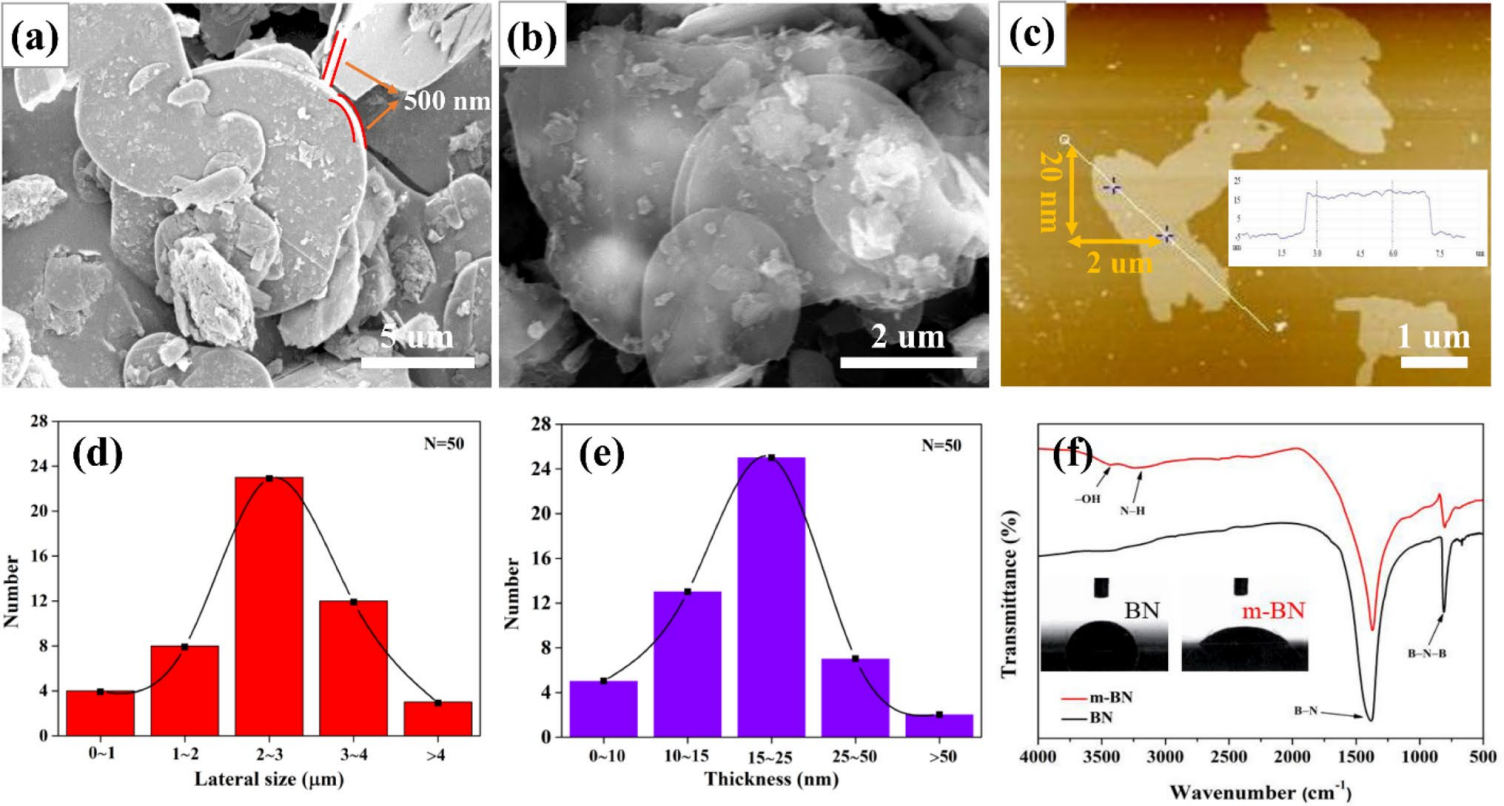
Characterizations of original BN and m-BN. **a, b** are SEM images of pristine BN and m-BN. **c** AFM image, **d** diameter length and **e** thickness distribution of m-BN. **f** FT-IR analysis of BN and m-BN. Insets are the corresponding contact angle test results

The original BN has chemical stability and large thickness, which limits its dispersibility in the organic matrix and the improvement of the overall TC [53]. To illustrate the successful stripping and modification of BN, the microscopic morphology and element bond analysis were tested by using SEM, AFM and FT-IR, respectively. Figure 1a shows the platelet morphology of the original BN, the uneven lateral size (about 5–10 μm) and the thickness of about 500 nm can be clearly observed. After modified by ball milling with boric acid (BA), the obtained m-BN presents a thin and transparent morphology (Fig. 1b). Taking 50 pieces of m-BN flakes for further observed with an atomic force microscope, from Fig. 1c–e, it is found that the m-BN with an average lateral size of 2–3 μm and a thickness of about 20 nm.

The mechanism of BN modification can be found in FT-IR spectra as shown in Fig. 1f. Except for two characteristic peaks of B-N stretching vibration (1375 cm^−1^) and B-N-B bending vibration (819 cm^−1^) about original BN, m-BN exhibits two other different broad absorptions at 3420 and 3230 cm^−1^, which correspond to the –OH and N–H stretching vibration [54, 55]. The acquisition of hydrophilic groups is due to the high-speed mechanical shearing action that makes the N atoms and B atoms in an active state to react with BA [56]. The increase in hydrophilicity of m-BN is verified by the fact that the contact angle of m-BN is significantly smaller than that of BN in the illustration.

**Fig. 2:**
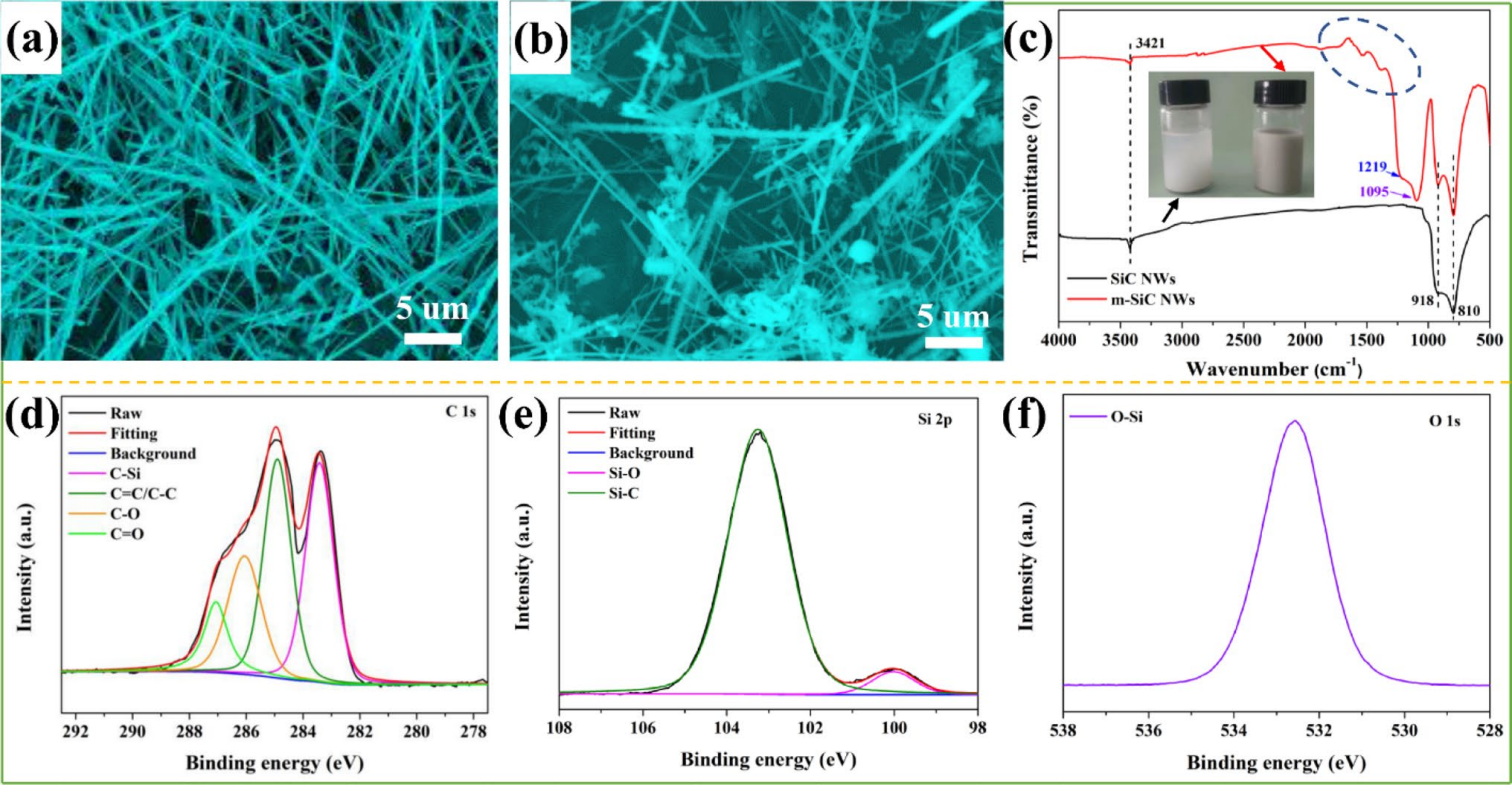
Characterizations of commercial SiC NW_S_ and m-SiC NW_S_. **a, b** are SEM images of commercial SiC NW_S_ and m-SiC NW_S_. **c** FT-IR spectra of SiC NW_S_ and m-SiC NW_S_. High-resolution XPS analysis of **d** C 1*s*, **e** Si 2*p* and **f** O 1*s* of m-SiC NW_S_

In order to reduce the interface thermal resistance between SiC NWs and BN, SiC NWs are treated by high-temperature oxidation. According to the SEM image in Fig. 2a, the untreated SiC NW_S_ have a rod-like structure with a length of about 25 μm and have a smooth surface without impurities. After calcination at 1300 °C, the surface of the m-SiC NW_S_ becomes rough and has granular aggregates (Fig. 2b). As shown in Fig. 2c, the FT-IR characteristic peaks at around 810 and 918 cm^−1^ are attributed to the C–Si stretching vibration of SiC NWs [57]. The peak at around 3421 cm^−1^ belongs to the –OH group in the water absorbed on the surface of the sample. After modification, the new peaks of Si–O–Si and C–O–Si groups at 1095 and 1219 cm^−1^ appear on m-SiC NWs [58]. Furthermore, a series of peaks located at around of 1500 cm^−1^ (inside the blue dashed circle) are ascribed to the stretching vibration of C–O and C=O [59].

Figure 2d–f presents the XPS high-resolution spectra of C, Si and O elements to further confirm the linking of functional groups on m-SiC NW_S_. From Fig. S1, the C 1*s* spectrum of commercial SiC NW_S_ shows three fitting peaks at 283.4, 284.9 and 286.4 eV corresponding to C–Si, C=C/C–C and C-O, respectively [60]. While the C 1*s* spectrum of m-SiC NW_S_ not only contains the above fitting peaks, but also emerges a C=O bond at 287.1 eV. More importantly, after modification, the peak attributed to C=C/C–C gets stronger. In addition, except for the Si–C bond at 102.8 eV in the Si 2*p* spectrum, an obvious fitting peak found in m-SiC NW_S_ at 100.3 eV corresponds to Si–O bond. [61, 62]. Only one main fitting peak of O 1* s* belongs to the O–Si bonding at 532.2 eV. From the above FT-IR and XPS results, it can be inferred that multi-oxygen-containing functional group (Si–O–Si, C–O–Si and C=O) were formed on the surface of m-SiC NW_S_ after calcination treatment [63]. These valence bond structures not only enhance the dispersion of m-SiC NW_S_ in the matrix (from the illustration in Fig. 2c, compared to the commercial SiC NW_S_, the aqueous dispersion of m-SiC NW_S_ did not show delamination after standing for 24 h), but also provide a theoretical support for m-SiC NW_S_ connecting m-BN to form thermally conductive paths.

### Fabrication and Characterization of CA/m-SiC/m-BN and CA/m-SiC/m-BN/EP

**Fig. 3:**
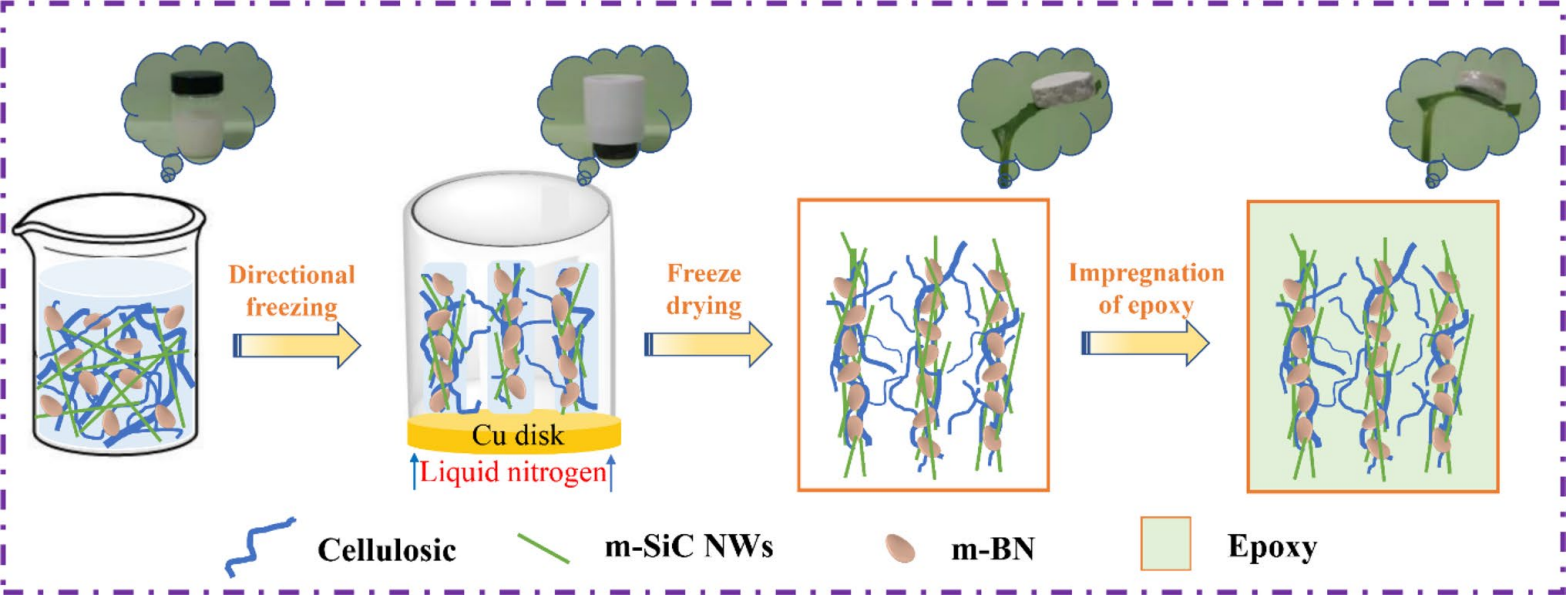
Schematic diagram of the preparation of CA/m-SiC/m-BN/EP

As shown in Fig. 3, the preparation of CA/m-SiC/m-BN/EP can be summarized in the following four processes: firstly, the preparation of a paste dispersion of cellulose/m-SiC/m-BN; then, the mixed dispersion is cross-linked in a mold to form m-SiC/m-BN cellulose hydrogel, which undergoes a directional freezing in a liquid nitrogen environment [64]; Next, the m-SiC/m-BN cellulose hydrogel is freeze-dried to become m-SiC/m-BN cellulose aerogel (CA/m-SiC/m-BN); Finally, CA/m-SiC/m-BN/EP is obtained by impregnating the CA/m-SiC/m-BN aerogel in EP with the aid of a vacuum system. In addition, it can be seen from the vignette that CA/m-SiC/m-BN is lightweight, and even the final CA/m-SiC/m-BN/EP composite can also be placed steadily on the tiny grass branches.

**Fig. 4:**
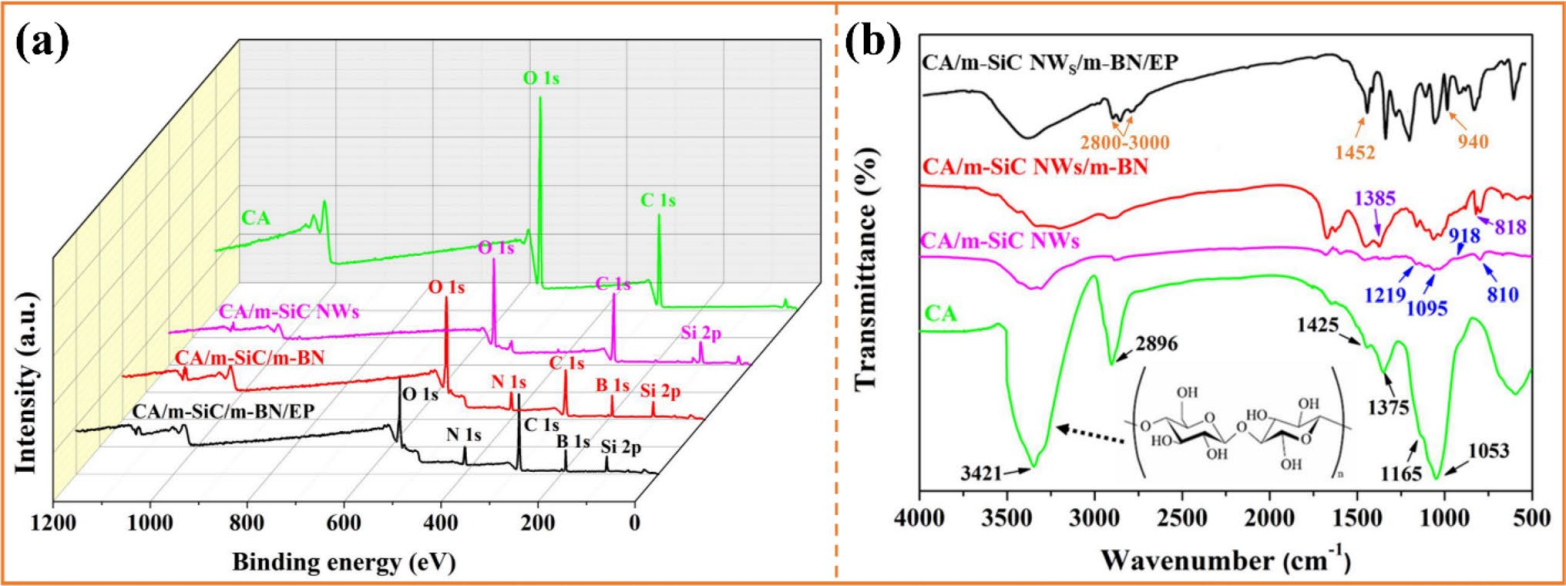
**a** XPS and **b** FT-IR spectra of CA, CA/m-SiC NW_S_, CA/m-SiC NW_S_/m-BN and CA/m-SiC NW_S_/m-BN/EP

Figure 4 presents the XPS and FT-IR spectra of cellulose aerogel (CA), CA/m-SiC NW_S_, CA/m-SiC/m-BN and CA/m-SiC/m-BN/EP to further confirm the covalent connection. Specifically, the binding energy peaks of different elements contained in CA, SiC NW_S_ and BN were observed on the hybrid spectrum of CA/m-SiC/m-BN and CA/m-SiC/m-BN/EP (Fig. 4a). As shown in Fig. 4b, the observed peak for pure CA centered at 3421, 1375, 1165 and 1053 cm^−1^ correspond to the –OH stretching vibration, –OH bending vibration, –CO antisymmetric bridge stretching vibration and –CO–C vibration of pyranoid ring skeleton, respectively [37, 65]. In addition, the bands at 2896 and 1425 cm^−1^ belong to the absorption peak of the –CH stretching vibration on the pyranoid ring and the branched chains, respectively. After the introduction of m-SiC NW_S_ and m-BN into cellulose, their characteristic peaks successively appeared on the infrared spectra of CA/m-SiC NW_S_ and CA/m-SiC/m-BN, which corresponded to the results in Figs. 1f and 2c. Furthermore, the FT-IR spectrum of the final product CA/m-SiC/m-BN/EP not only contains all the above characteristic peaks, but also adds the obvious absorption peaks of the EP matrix. The bands at 2830–3000, 1452 and 940 cm^−1^ correspond to the stretching vibration of –CH/–CH_2_/–CH_3_, the stretching vibration peak of benzene ring and the characteristic absorption peak of epoxy group (–CH(O)CH–), respectively. It is worth mentioning that the cellulose with polyhydroxy group (inset in Fig. 4b), the m-SiC NW_S_ with multi-oxygen-containing functional group and m-BN with N–H group are tightly combined together relying on the hydrogen bond between these groups. Thus, a stable and continuous thermally conductive structure is formed in CA/m-SiC/m-BN/EP [66].

**Fig. 5:**
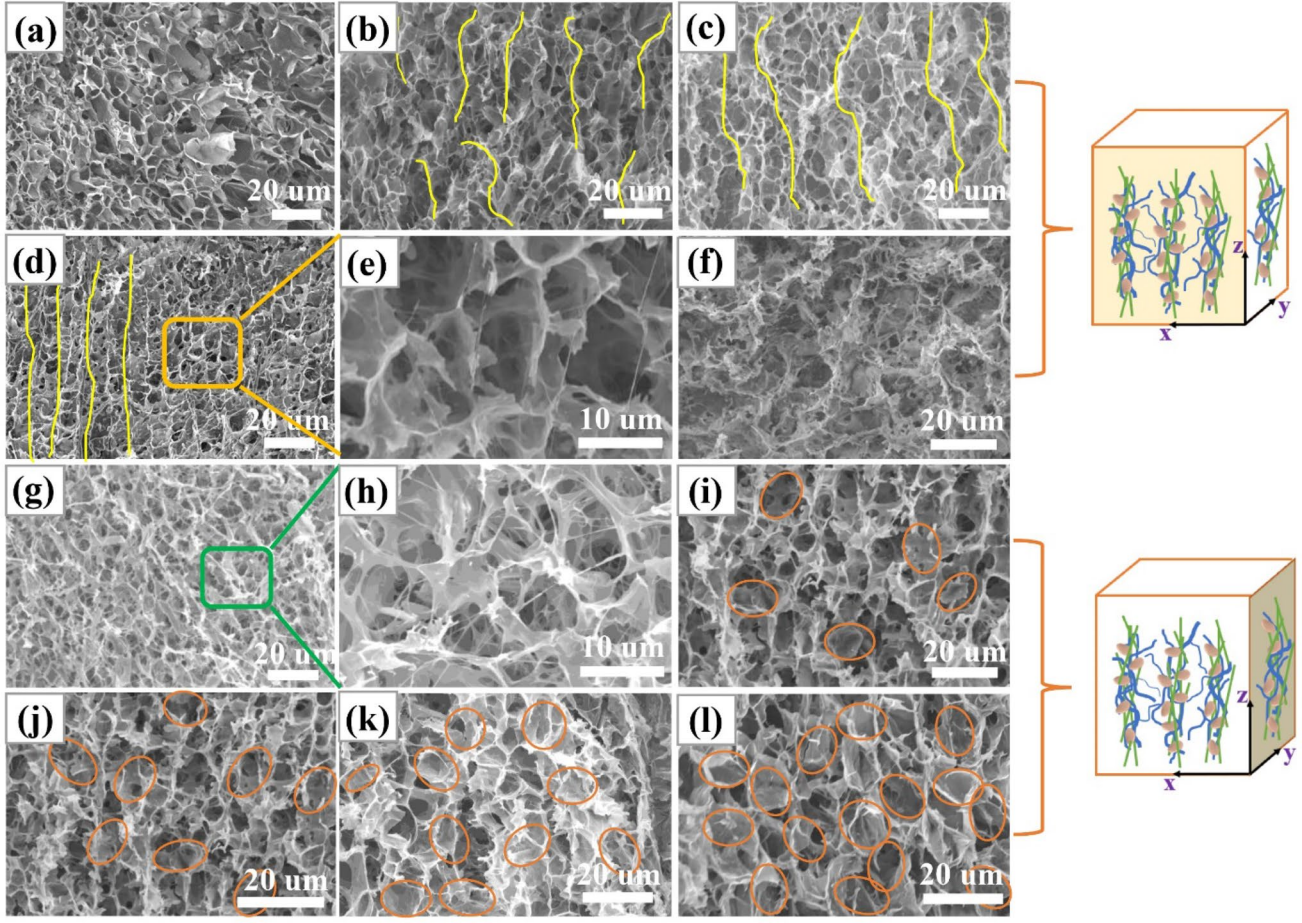
SEM images of CA/m-SiC NW_S_/m-BN with different contents of m-SiC NW_S_ and GO and m-BN. **a–d** and **f** are the section morphologies of CA/1.8 m-BN, CA/0.03 m-SiC/1.8 m-BN, CA/0.06 m-SiC/1.8 m-BN, CA/0.10 m-SiC/1.8 m-BN and CA/0.13 m-SiC/1.8 m-BN skeleton in *x*–*z* plane. **e** is local enlarged drawing of d. **g**, **i–l** are the section morphologies of CA/0.10 m-SiC, CA/0.10 m-SiC/0.5 m-BN, CA/0.10 m-SiC/1.0 m-BN, CA/0.10 m-SiC/1.5 m-BN and CA/0.10 m-SiC/1.8 m-BN skeleton in *x*–*y* plane. **h** is local enlarged drawing of g

The microstructure of the thermally conductive filler has a significant effect on the TC of the composite. In this work, the microstructure of CA/m-SiC/m-BN is affected by the content of m-SiC NW_S_ and m-BN. From Fig. S2, pure cellulose presents a unique porous network structure with a pore diameter of about 10 μm [67]. The influence of m-SiC NW_S_ on the overall microstructure was studied by changing its content (0.00, 0.03, 0.06, 0.10 and 0.13 g) when the mass of m-BN in the aerogel was controlled at 1.8 g. As shown in Fig. 5a, when m-SiC NW_S_ is not introduced, m-BN in CA/1.8 m-BN is embedded on the hole wall of the CA. With the addition of m-SiC NW_S_ in different masses and assisted by directional freezing, the CA/m-SiC/m-BN skeleton presents a vertically oriented structure in the *x*–*z* plane (Fig. 5b–d, f). Especially, when the mass of m-SiC NW_S_ is 0.10 g, CA/0.10 m-SiC/1.8 m-BN shows a high orientation along the ice growth direction. From the enlarged SEM image of Fig. 5e, it can be clearly seen that the m-SiC NW_S_ are arranged in a vertical direction. However, as the mass of SiC NWs continues to increase to 0.13 g, SiC NWs are entangled with each other to limit the vertical growth of ice crystals, resulting in a porous network structure with different pore sizes.

The influence of m-BN on the overall microstructure was studied by changing its content (0.0, 0.5, 1.0, 1.5 and 1.8 g) when the mass of SiC NWs in the aerogel was controlled at 0.10 g. From Fig. 5g–h, CA/0.10 m-SiC shows a network structure of cellulose and m-SiC NW_S_ connected without the participation of m-BN. In order to more intuitively show the influence of different contents of m-BN on the overall morphology, the structural characterizations of CA/m-SiC/m-BN skeleton in the *x*–*y* plane are shown in Fig. 5i–l. It can be seen that the embedded m-BN in the cellulose increases with increasing its mass. When the mass of m-BN reaches to 1.8 g, the m-BN starts to connect to each other, which is the best content for the construction of thermal conduction path, because excessive BN will aggregate and then increase the interface thermal resistance. More importantly, combined with Fig. 5d, l, it is easy to observe that m-SiC NW_S_ and m-BN form a continuous thermally conductive network structure on the pore wall of CA, which is conducive to the rapid transfer of phonons between the two fillers.

In order to explore the influence of the microscopic morphology of the composite on the thermally conductive properties, the morphology analysis of the EP-based composites is further carried out. As can be seen from Fig. S3a, the cross-sectional morphology of CA/0.10 m-SiC/EP without m-BN shows a stripe structure with single crack direction and vertical extension, and m-SiC NW_S_ can be evenly distributed in EP, which is attributed to their good interfacial compatibility. Similarly, from Fig. S3b, the cross-sectional morphology of CA/1.8 m-BN/EP without m-SiC NW_S_ shows that m-BN is uniformly distributed in EP. For the CA/0.10 m-SiC/1.8 m-BN/EP_blend_ obtained by simple blending, the cross-sectional appearance has no regular and fixed orientation (Fig. S3c). For the optimal sample CA/0.10 m-SiC/1.8 m-BN/EP, an obvious vertical stratification can be observed (Fig. S3d). These results further confirm the successful acquisition of EP-based composites with a vertically oriented thermally conductive network. In addition, the EP can penetrate into the 3D network since the modification of the fillers enhances the interfacial interaction. Therefore, this well-arranged vertical structure can provide an efficient heat transfer channel in the direction of the through-plane, resulting in excellent anisotropic TC [68].

### Anisotropic Thermal Properties of CA/m-SiC/m-BN/EP

**Fig. 6:**
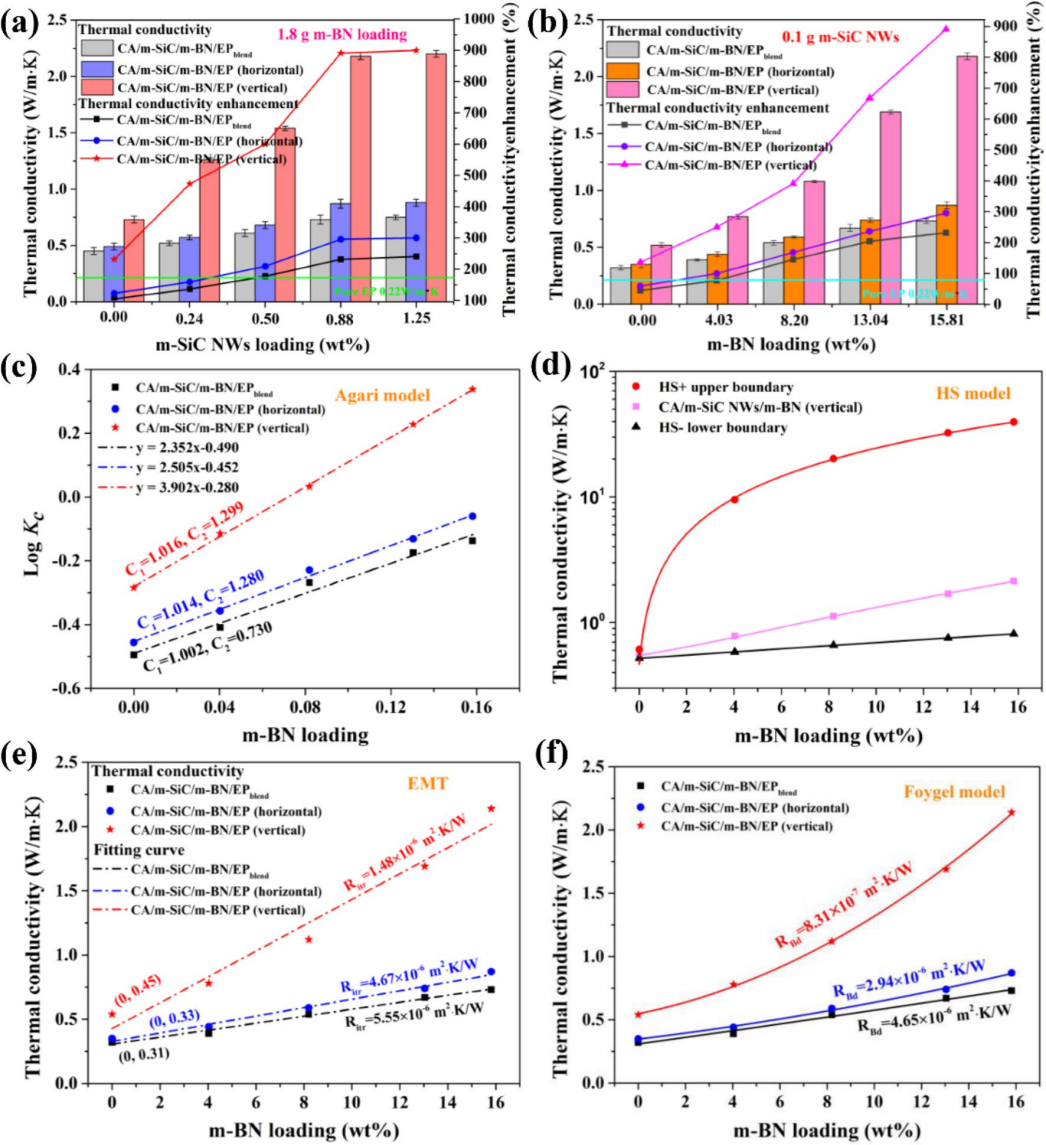
Anisotropic thermal properties analysis of CA/m-SiC/m-BN/EP. **a** Thermal conductivities and thermal conductivity enhancements of CA/m-SiC/m-BN/EP and CA/m-SiC/m-BN/EP_blend_ composites with different m-SiC NW_S_ contents at the m-BN mass of 1.8 g. **b** Thermal conductivities and thermal conductivity enhancements of CA/m-SiC/m-BN/EP and CA/m-SiC/m-BN/EP_blend_ composites with different m-BN contents at the m-SiC NW_S_ mass of 0.10 g. **c** Agari model fitting lines, **d** Hashin–Shtrikman (HS) model fitting lines, **e** EMT model fitting lines and **f** Foygel’s theory fitting lines of CA/m-SiC/m-BN/EP and CA/m-SiC/m-BN/EP_blend_ composites

Combined with the thermal gravimetric analyzer, the density and filler loadings of different samples are presented in Tables S1 and S2. Figure 6a, b presents the TC of CA/m-SiC/m-BN/EP composites in both vertical plane and horizontal plane directions with different m-SiC NW_S_ and m-BN contents. It can be seen that the TC of CA/m-SiC/m-BN/EP and CA/m-SiC/m-BN/EP_blend_ composites increased with increasing the content of m-SiC NW_S_ and m-BN, and the existence of the unique thermally conductive network results in a significant enhancement of TC in both directions. For example, whether it is CA/1.8 m-BN/EP and CA/0.10 m-SiC/EP with a single filler, or CA/m-SiC/m-BN/EP with a composite filler, they all perform higher TC than pure EP (0.22 W m^−1^ K^−1^) in both vertical plane and horizontal plane. Moreover, at a specific content of m-SiC NW_S_ and m-BN, the TC in both planes for the composite with unique network structure is better than that of the composites with a random dispersion [69]. It is worth noting that in Fig. 6a, the TC of the EP-based composite has basically not changed with the content of m-SiC NW_S_ increased from 0.88 to 1.25 wt% (i.e., 0.10–0.13 g). This is mainly attributed to excessive m-SiC NW_S_ which are entangled with each other (Fig. 5f) and prevent further improvement of TC. In order to visually highlight the superior thermally conductive properties of the composite under the optimal filler content, thermal conductivity enhancement (TCE) was adopted using Eq. (1) [70]:

1 $$ {\text{TCE}} = \frac{{K_{{\text{c}}} - K_{{\text{m}}} }}{{K_{{\text{m}}} }} \times 100\% $$

where *K*_c_ and *K*_m_ are the TC of EP-based composites and pure EP. After calculation, the TCE of the CA/0.10 m-SiC/1.8 m-BN/EP (*K*_c_ = 2.21 W m^−1^ K^−1^) composite reached 890.9% at the total filler loading of 16.69 wt%, exhibiting excellent TC enhancement performance. In summary, the appropriate contents of m-SiC NW_S_ and m-BN in CA/m-SiC/m-BN/EP are 0.88 wt% (0.10 g) and 15.81 wt% (1.8 g), respectively.

The TC of the EP-based composites is theoretically described by classic Agari and Hashin–Shtrikman (HS) models, which are often used to test the filler distribution in the specific network. In view of the relatively low content of m-SiC NWS (about 0.24–0.88 wt% concluded from Tables S1 and S2) in CA/m-SiC/m-BN/EP, the filler m-BN contributes significantly better to the TC of the composite than m-SiC as shown in Fig. 6a, b. Therefore, in this work, m-SiC/EP is regarded as a matrix and m-BN is used as a thermally conductive filler to simplify the simulation structure. The Agari model is shown as Eq. (2) [71]:

2 $$ \log K_{{\text{C}}} = \varphi C_{2} \log K_{f} + \left( {1 - \varphi } \right)\log \left( {C_{1} K_{{\text{m}}} } \right) $$

where *K*_c_, *K*_*f*_ and *K*_m_ represent the TC of EP-based composites, m-BN and matrix (m-SiC/EP), respectively. Here, *K*_*f*_ is 350 W m^−1^ K^−1^ in horizontal and 600 W m^−1^ K^−1^ in the vertical direction, and 30 W m^−1^ K^−1^ in random dispersion. *K*_m_ is 0.35 W m^−1^ K^−1^ in horizontal and 0.52 W m^−1^ K^−1^ in the vertical direction, and 0.32 W m^−1^ K^−1^ in random dispersion. *φ* is the weight fraction of m-BN filler. *C*_1_ is the influence parameter of filler on the secondary structure of polymer matrix. *C*_2_ measures how easily the filler can form a thermally conductive network [72]. From Fig. 6c, compared with CA/m-SiC/m-BN/EP_blend_ through a simple blending (random dispersion) method, CA/m-SiC/m-BN/EP obtained by the ice template method has larger *C*_1_ and *C*_2_ after simulation calculation on the vertical and horizontal planes. This indicates that an ideal crystal structure and heat conduction paths are formed inside CA/m-SiC/m-BN/EP [73], especially in the vertical direction.

In order to further highlight the unique vertical net structure formed inside CA/m-SiC/m-BN/EP, the Hashin–Shtrikman (HS) model is introduced as Eqs. (3, 4) [74]:

3 $$ K^{{{\text{HS}} + }} = K_{f} \frac{{2K_{f} + K_{{\text{m}}} - 2\varphi_{{\text{m}}} \left( {K_{f} - K_{{\text{m}}} } \right)}}{{2K_{f} + K_{{\text{m}}} + \varphi_{{\text{m}}} \left( {K_{f} - K_{{\text{m}}} } \right)}} $$

4 $$ K^{{{\text{HS}} - }} = K_{m} \frac{{2K_{{\text{m}}} + K_{f} - 2\varphi_{{\text{f}}} \left( {K_{{\text{m}}} - K_{f} } \right)}}{{2K_{{\text{m}}} + K_{f} + \varphi_{{\text{f}}} \left( {K_{{\text{m}}} - K_{f} } \right)}} $$

where the *K*^HS+^ (upper boundary) refers to the matrix that is completely surrounded by filler; *K*^HS+^ (lower boundary) indicates that the fillers are completely separated by matrix. *K*_*f*_ and *K*_m_ represent the TC of m-BN and matrix (m-SiC/EP), In that case, *K*_*f*_ takes a value of 600 W m^−1^ K^−1^ and *K*_m_ takes a value of 0.52 W m^−1^ K^−1^. *φ*_m_ and *φ*_f_ are the weight fraction of matrix and filler. Figure 6d presents the simulation results of anisotropic TC in accordance with HS model. Obviously, the measured TC stays between the upper and lower boundaries, which meets the fitting result of the HS model. The appearance of the approximate logarithmic fitting curve of the HS + upper boundary shows that the construction of the heat conduction paths in this experiment can achieve a greater impact on the overall TC with a low m-BN content.

In practical application, the interfacial thermal resistance between the filler/matrix and filler/filler in the thermally conductive composites is the key factor affecting the heat transfer process [26]. Therefore, the effective medium theory (EMT) and the Foygel theory are used to model and analyze the interfacial thermal resistance in the samples. EMT is used to fit the relationship between the interface thermal resistance from filler/matrix and the TC as shown in Eq. (5) [75]:

5 $$ K_{{\text{c}}} = K_{{\text{m}}} + \frac{{a\varphi_{{\text{f}}} K_{{\text{m}}} }}{{3\left( {a + \frac{{R_{{{\text{itr}}}} K_{f} }}{L}} \right)}} $$

where *K*_c_, *K*_*f*_ and *K*_m_ are the TC of composite, m-BN and matrix (m-SiC/EP), respectively. *φ*_f_ is the weight fraction of m-BN, *R*_itr_ is the interfacial thermal resistance at m-BN/matrix interface, *L* and* a* are the average diameter length and diameter-thickness ratio of m-BN, respectively. The corresponding fitting results are shown in Fig. 6e and the values of *R*_itr_ for CA/m-SiC/m-BN/EP in both vertical and horizontal directions and randomly dispersed CA/m-SiC/m-BN/EP_blend_ composites are 1.48 × 10^–6^, 4.67 × 10^–6^, and 5.55 × 10^–6^ m^2^ KW^−1^, respectively. This result clearly proves that the successful modification of m-BN enhanced its interaction with the matrix. In addition, *R*_itr_ in vertical direction is lower than horizontal direction, which indicates that m-BN can also be tightly bonded to m-SiC (as the matrix) in the vertical direction.

The Foygel model is applied to simulates and calculate the thermal boundary resistance between fillers, as shown by Eqs. (6, 7) [76]:

6 $$ K_{{\text{c}}} = K_{{\text{m}}} + K_{0} \left[ {\frac{{\varphi_{{\text{f}}} - \varphi_{{\text{c}}} }}{{1 - \varphi_{{\text{c}}} }}} \right]^{\beta } $$

7 $$ R_{{{\text{Bd}}}} = \frac{1}{{K_{0} L\left( {\varphi_{{\text{c}}} } \right)^{\beta } }} $$

where *K*_c_ and *K*_m_ are the TC of composite and matrix, *K*_0_ is a pre-exponential factor related to the filler. *φ*_f_ is the weight fraction of m-BN, *φ*_c_ is the critical permeability content of the filler, *L* is the average diameter length of m-BN, β is related to the conductivity index of the filler, and *R*_Bd_ is the thermal boundary resistance at filler/filler interface. It can be seen from Fig. 6f that the *R*_Bd_ of CA/m-SiC/m-BN/EP in vertical direction is as low as 8.31 × 10^–7^ m^2^·K/W, which is nearly one order of magnitude lower than the results of horizontal direction (2.94 × 10^–6^ m^2^ K W^−1^) and random distribution (4.65 × 10^–6^ m^2^ K W^−1^). Furthermore, the appearance of an approximate exponential fitting curve in the vertical direction indicates that m-BN is arranged in an orderly manner and no stacking occurs in this direction. This further confirmed the advantages of CA/m-SiC/m-BN/EP with high-speed heat conduction channel in the vertical direction.

To demonstrate the advantage of CA/m-SiC/m-BN/EP obtained in this work in terms of thermally conductive performance, a comparison with previous similar studies is shown in Table S3. After comprehensive comparison, the CA/m-SiC/m-BN/EP exhibits the excellent TC at low content among the reported bulk composites.

**Fig. 7:**
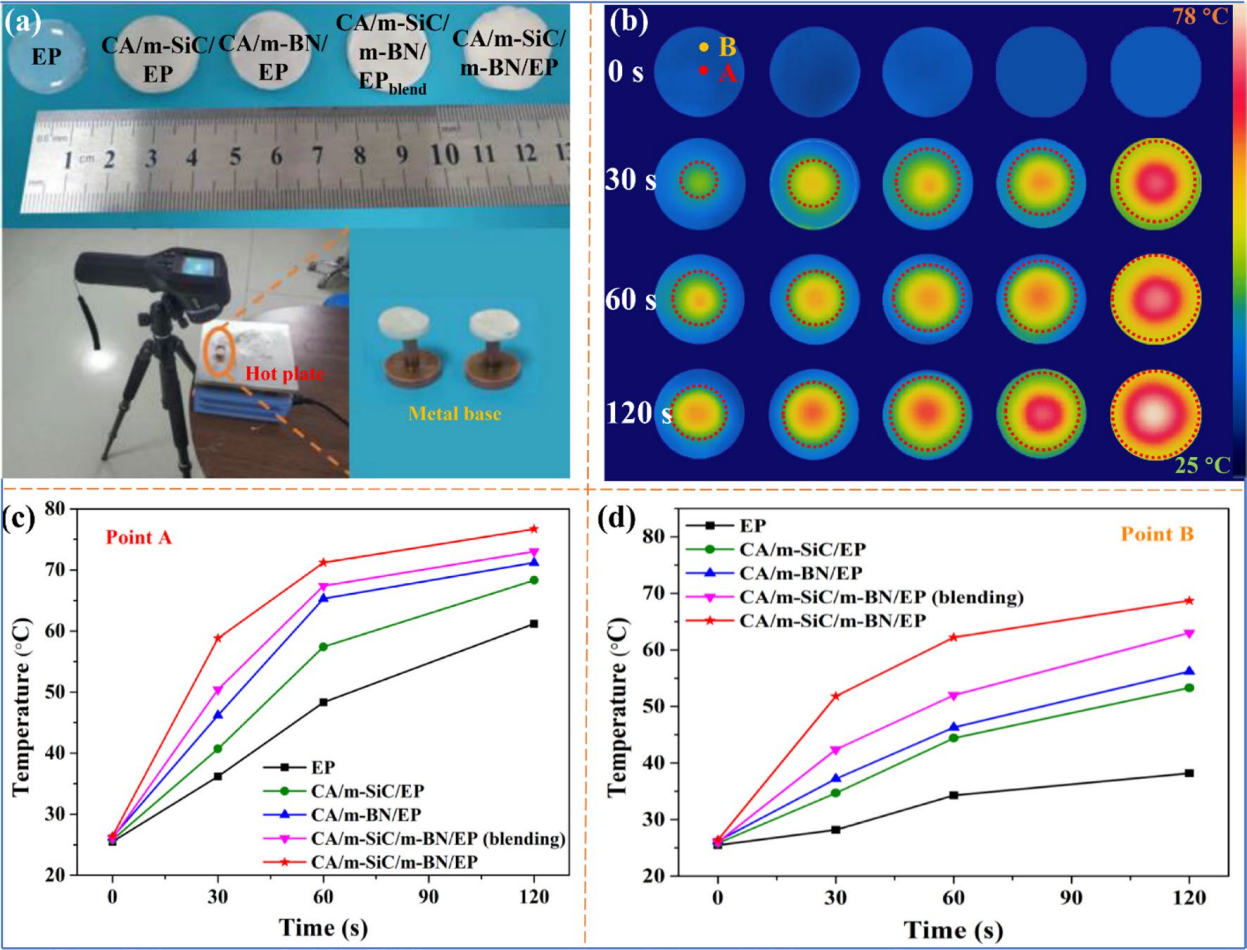
**a** Optical photographs of different EP-based composites and schematic design for thermal imaging test. **b** Infrared thermal images of different EP-based composites variation with heating time and **c–d** the temperature changes of A and B on the surface of EP-based composites with heating time

Infrared thermal imager was used to intuitively estimate the thermal management capability of different EP-based composites. Specifically, as shown in Fig. 7a, different samples with a diameter of 2 cm and a thickness of 3 mm are placed simultaneously on a hot plate set at 80 °C, and then, the infrared thermal imager is used to record the surface temperature changes over time. In order to verify that the TC of CA/m-SiC/m-BN/EP is anisotropic in two directions, the custom metal bases are used as the point heat source for the sample in the experimental design. It is worth noting that two test points A and B are selected on the sample surface during the experiment, as shown in Fig. 7b; point A is used to test the temperature change of the sample center with the point heat source in the vertical direction, while point B is used to reflect the thermal conduction capability of the sample in the horizontal direction. It is found that the surface temperature of different samples in the following order: CA/m-SiC/m-BN/EP > CA/m-SiC/m-BN/EP_blend_ > CA/m-BN/EP > CA/m-SiC/EP > EP, this result is consistent with the order of TC obtained in Fig. 6a, b. Obviously, the surface temperature of CA/m-SiC/m-BN/EP at point A shows a fastest increasing with time compared to other samples. The specific temperature change with time of point A recorded by a computer is shown in Fig. 7c. After 120 s, the center temperature of CA/m-SiC/m-BN/EP is as high as 76.7 °C, which is close to the temperature of hot plate. Not only that, as shown in Fig. 7d, the sample CA/m-SiC/m-BN/EP also showed excellent heat transfer efficiency at point B close to the edge (the temperature is about 67.2 °C after 120 s), which shows that CA/m-SiC/m-BN/EP has high thermal conductivity along both vertical and horizontal directions, thereby forming a heat dissipation area with a larger radius [77].

### Thermal Conduction Mechanism of Different EP-Based Composites

**Fig. 8:**
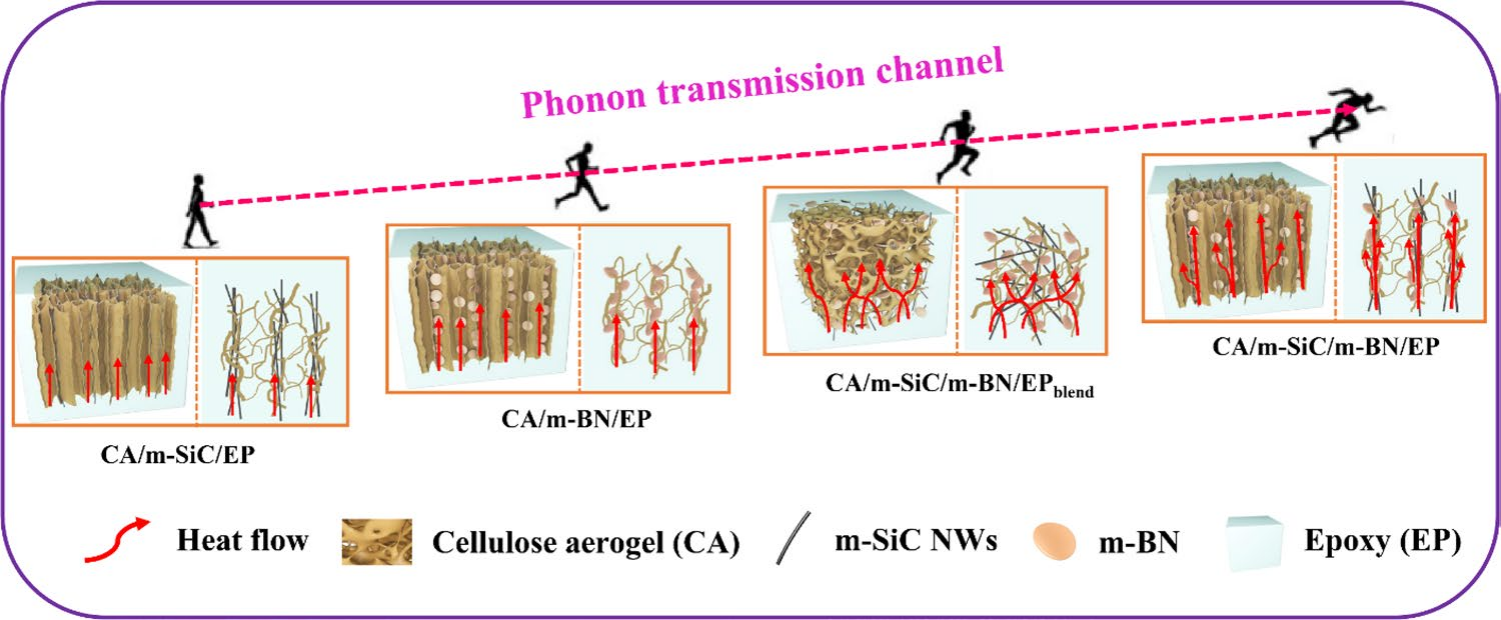
Schematic diagram of heat flow of different EP-based composites along the vertical direction

According to the morphology characterization and TC test results of different samples, the heat flow transmission path can be reasonably simulated. In addition, the packaging material is generally coated on the surface of the microelectronic device, so here we focus on the comparison and analysis of the thermally conductive mechanism of different samples in the vertical direction. As we all know, phonons are the collective modulus of the vibrations of mutually coupled atomic lattice systems and are the main thermal energy carriers of polymer-based materials. Therefore, constructing efficient phonon transport channel is the key to improve the TC of thermal interface composites. As shown in Fig. 8, for CA/m-SiC/EP and CA/m-BN/EP with a single filler, the phonon transmission channel mainly relies on the vertically oriented m-SiC NWs and m-BN network structures, respectively. Since the in-plane TC of BN (600 W m^−1^ K^−1^)) is higher than the in-line thermal conductivity of m-SiC NWs (100 W m^−1^ K^−1^), CA/m-BN/EP exhibits a faster heat flow transfer rate. At the optimal filler content, the TC of CA/m-SiC/m-BN/EP_blend_ with dual fillers randomly dispersed is higher than that of CA/m-BN/EP and CA/m-SiC/EP, which is mainly due to the fact that more phonon transmission paths can be formed inside the structure of CA/m-SiC/m-BN/EP_blend_ [78]. When m-SiC NWs and m-BN form a synergistic vertical network structure in CA/m-SiC/m-BN/EP, resulting in a faster heat flow transfer compared to other samples. Hence, the vertically aligned networks composed of interconnected m-SiC NWs and m-BN heat conductive paths play a critical role in the phonon transmission, which can significantly improve the TC of the composite in the vertical direction [79].

### Electrical Insulative Properties of Different EP-Based Composites

**Fig. 9:**
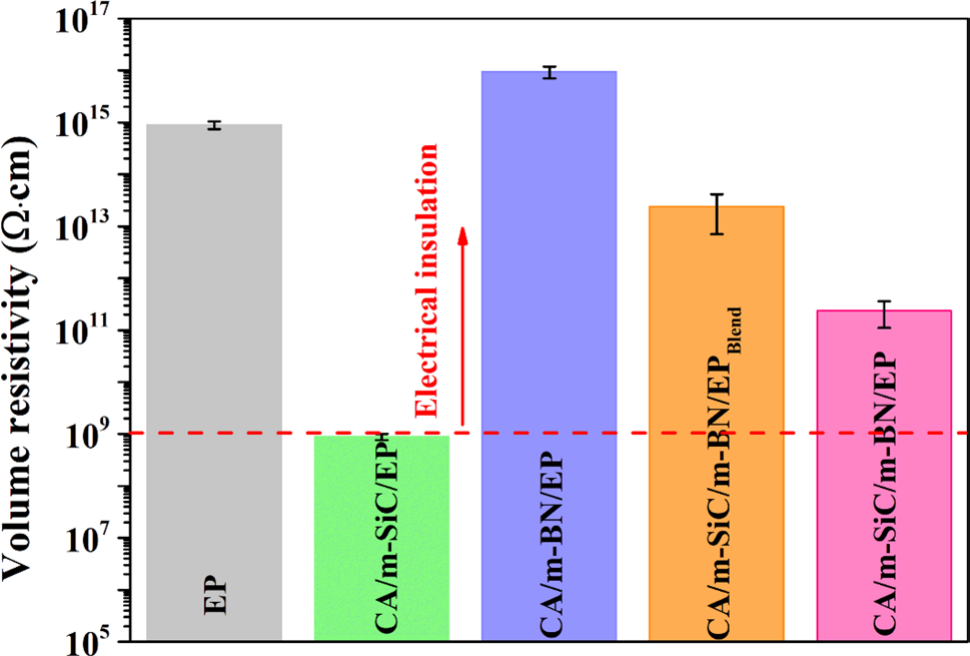
The volume resistivity of pure EP and different EP-based composites in the vertical direction

Electrical insulation is the basic feature of electronic packaging materials [4]. During the experiment, conductive glue was applied on both sides of the tested sample and the experimental results are shown in Fig. 9. It can be seen that compared with pure EP (8.97 × 10^14^ Ω·cm), the resistivity of CA/m-SiC/EP (8.87 × 10^8^ Ω·cm) decreases sharply, while the resistivity of CA/m-BN/EP (9.47 × 10^15^ Ω·cm) increases slightly, which is mainly attributed to the linear semiconductor characteristics of m-SiC NW_S_ and the high electrical insulation of BN [80]. For the CA/m-SiC/m-BN/EP_blend_ obtained by random dispersion, there is no consistent linear direction despite the participation of m-SiC NW_S_, so its resistivity is mainly influenced by EP, which is about 2.42 × 10^13^ Ω·cm. It is worth mentioning that the resistivity of CA/m-SiC/m-BN/EP (2.35 × 10^11^ Ω·cm) with a vertical network structure decreases compared with that of pure EP and CA/m-SiC/m-BN/EP_blend_, but it is much higher than the theoretical critical resistivity (10^10^ Ω·cm) [25], combined with the high resistivity value of CA/m-BN/EP, this is mainly due to the high insulation of m-BN playing an important role in this special structure. It is convinced that the thermal management composite CA/m-SiC/m-BN/EP obtained in this work has a great potential for application in the field of microelectronic packaging.

### Dielectric and Electromagnetic Wave Absorption Performances of Different Samples

**Fig. 10:**
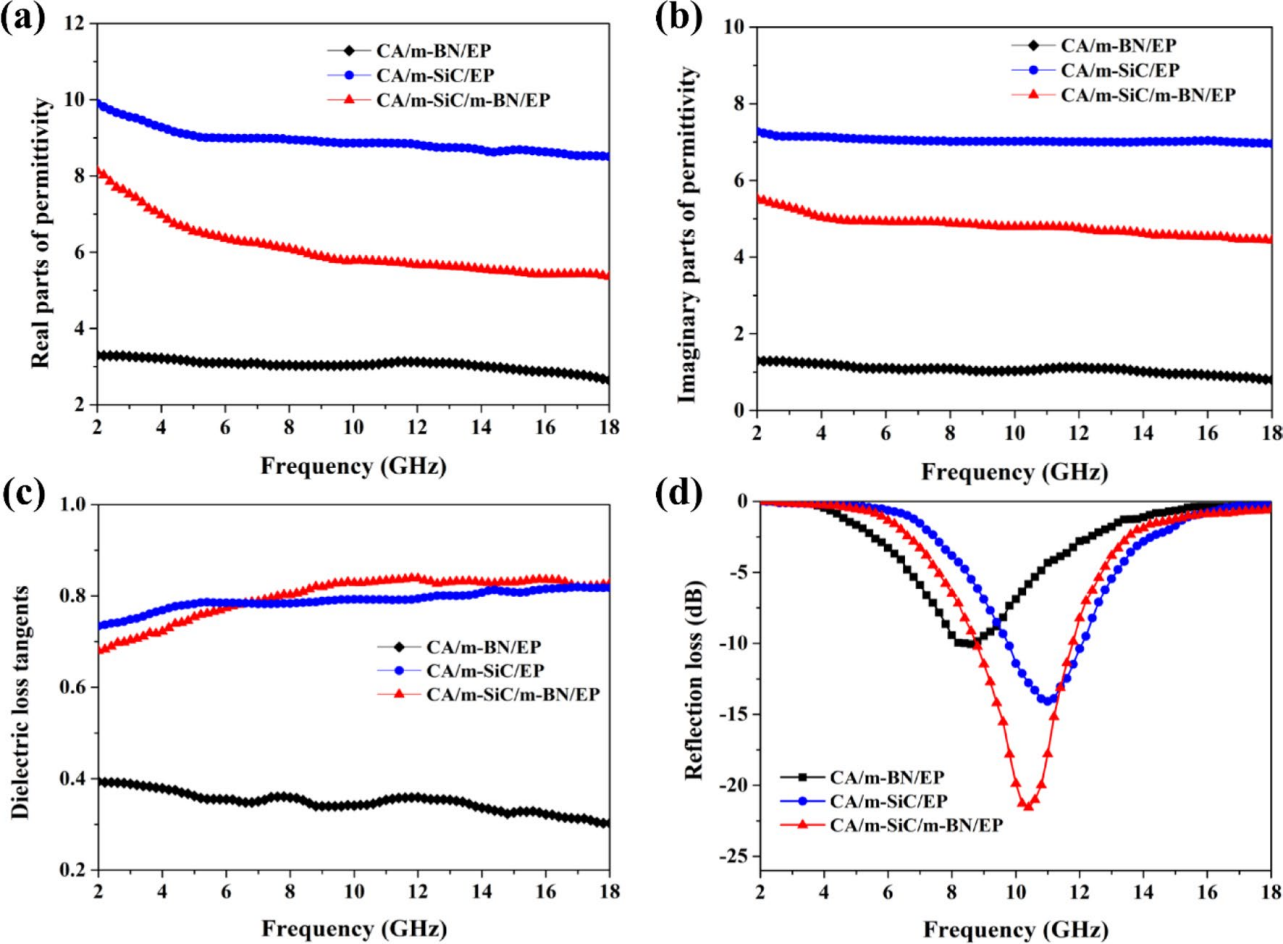
**a** Real parts and **b** imaginary parts of the complex permittivities. **c** dielectric loss tangents and **d** reflection loss of the CA/m-SiC/EP, CA/m-BN/EP and CA/m-SiC/m-BN/EP

SiC NWs are a very important wide-bandgap semiconductor material and have a high dielectric loss capability, so it shows excellent electromagnetic wave absorption potential [81]. In addition, EP with high resistivity (8.97 × 10^14^ Ω·cm) has almost no dielectric response, so the electromagnetic wave absorption capacity of different EP-based composites (CA/m-BN/EP, CA/m-SiC/EP and CA/m-SiC/m-BN/EP) mainly depends on the composition and structural framework of the internal filler. Generally speaking, the electromagnetic wave absorption capacity of non-magnetic dielectric materials is directly determined by the real (*ε*′) and imaginary (*ε*″) parts of the complex permittivity, *ε*′ and *ε*″ represent the ability to store and dissipate electromagnetic waves, respectively [82, 83]. The obtained *ε*′, *ε*″ and the corresponding dielectric loss tangents (tan *δ*_*ε*_ = *ε*″/*ε*′) are displayed in Fig. 10a–c. According to dielectric theory, the dielectric loss parameter ε′ for dielectric materials, while ε″ is attributed to the electrical conductivity. And the fast movement of electrons in high-conductivity fillers can also promote polarization. Based on the conductivity of m-SiC is much higher than that of insulating m-BN, therefore, the CA/m-BN/EP exhibits low *ε*′, *ε*″, and tanδ values, namely, 2.6–3.3, 0.8–1.3, and 0.3–0.4, respectively. Compared with CA/m-BN/EP, the *ε*′, *ε*″, and tan *δ*_*ε*_ values of CA/m-SiC/EP show significant enhancement to 8.5–9.9, 7.0–7.3, and 0.7–0.8, respectively. In addition, tan *δ*_*ε*_ indicates the ability of material to convert electromagnetic waves into other forms of energy (most of which are converted into heat), thereby achieving the dissipation of microwave energy, and a high tan *δ*_*ε*_ value indicates the strong ability to absorb electromagnetic waves [84]. It can be seen from Fig. 10c that CA/m-SiC/m-BN/EP not only has excellent microwave absorption capacity, but also it can convert the absorbed electromagnetic wave energy into heat for effective evacuation through the vertical heat conduction paths according to the aforementioned analysis of thermally conductive properties [85].

To evaluate the microwave absorption properties of different composites, the reflection loss (RL) values were calculated according to the transmit line theory by the following Eqs. (8, 9) [86]:

8 $$ {\text{RL}} = 20\log \left| {\frac{{Z_{{{\text{in}}}} - 1}}{{Z_{{{\text{in}}}} + 1}}} \right| $$

9 $$ Z_{{{\text{in}}}} = \sqrt {{\raise0.7ex\hbox{${\mu_{r} }$} \!\mathord{\left/ {\vphantom {{\mu_{r} } {\varepsilon_{r} }}}\right.\kern-\nulldelimiterspace} \!\lower0.7ex\hbox{${\varepsilon_{r} }$}}} \tanh \left[ {j\left( {\frac{2\pi fd}{c}} \right)\left( {\sqrt {\mu_{r} \varepsilon_{r} } } \right)} \right] $$

where *Z*_in_ is the input impedances of the composite, $\mu_{r}$ and $\varepsilon_{r}$ denote the complex permeability and complex permittivity, respectively, *f* represents the frequency of microwaves, *d* refers to the thickness of the composites and *c* is the velocity of light in vacuum.

Figure 10d shows the electromagnetic wave reflection loss curve of the three systems with a thickness of 3 mm. For the CA/m-SiC/m-BN/EP, the minimum reflection loss is − 21.5 dB at 10.4 GHz, and the effective absorption bandwidth (< − 10 dB) is from 8.8 to 11.6 GHz. According to Maxwell–Wagner polarization theory, this is mainly attributed to the vertical network structure formed by CA/m-SiC/m-BN in composite, which not only strengthens the effective interface (between m-SiC and m-BN) of interfacial polarization, but also enhances the microwave reflection paths [87]. In order to verify the excellent microwave absorption properties of the composites, the impedance matching between the materials and the incident microwaves was further explored. From Fig. S4, compared with CA/m-BN/EP, the microwave impedance of CA/m-SiC/EP and CA/m-SiC/m-BN/EP increases greatly because of their lower complex permittivity. In addition, CA/m-SiC/m-BN/EP exhibits better microwave absorption performance than CA/m-SiC/EP due to higher microwave impedance and almost the same dielectric loss.

## Conclusions

In summary, vertically aligned m-SiC NWs/m-BN cellulose aerogel (CA/m-SiC/m-BN) networks have been successfully constructed by ice template combined with directional freezing technology. CA/m-SiC/m-BN/EP composites were prepared by infiltrating the vertical networks with EP. In particular, the high-temperature calcination treatment of SiC NWs and the combined boric acid ball milling modification of BN not only improve the compatibility between the filler and matrix, but also reduce the thermal boundary resistance between the filler and filler, thereby effectively improving the thermal conductivity of the composite material. The CA/m-SiC/m-BN/EP composites exhibit a significantly enhanced TC of 2.21 W m^−1^ K^−1^ in vertical plane at a low filler loading of 16.69 wt%, which is increased by 890% compared to pure EP. In addition, from the analysis results of infrared thermal imaging, it is found that CA/m-SiC/m-BN/EP in the horizontal direction also exhibits a better thermally conductive performance than pure EP, CA/m-SiC/EP, CA/m-BN/EP and CA/m-SiC/m-BN/EP_blend_ with random dispersion. Furthermore, CA/m-SiC/m-BN/EP also has a superior volume resistivity of 2.35 × 10^11^ Ω·cm and a minimum reflection loss of − 21.5 dB. Therefore, the new EP-based composite synthesized in this work will have good application prospects in the fields of electronic packaging.

## Supplementary Information

Below is the link to the electronic supplementary material.

Supplementary file1 (PDF 650 KB)
