# Supplementary material for: Vertically Aligned Silicon Carbide Nanowires/Boron Nitride Cellulose Aerogel Networks Enhanced Thermal Conductivity and Electromagnetic Absorbing of Epoxy Composites
Source: Nanomicro Lett. 2022 Apr 30;14:118. doi: 10.1007/s40820-022-00863-z (PMC9056589; doi:10.1007/s40820-022-00863-z)
Supplement: Supplementary file 1 — Supplementary file1 (PDF 650 KB) [file 40820_2022_863_MOESM1_ESM.pdf]

Supporting Information for

Vertically Aligned Silicon Carbide Nanowires/Boron Nitride Cellulose Aerogel Networks Enhanced Thermal Conductivity and Electromagnetic Absorbing of Epoxy Composites

Duo Pan[1, 4], Gui Yang[1], Hala M. Abo-Dief[2], Jingwen Dong[1], Fengmei Su[1, *], Chuntai Liu[1], Yifan Li[3], Ben Bin Xu[3, *], Vignesh Murugadoss[4, 5], Nithesh Naik[7], Salah M. El-Bahy[8], Zeinhom M. El-Bahy[9], Minan Huang[4, 6], and Zhanhu Guo[4, *]

[1]Key Laboratory of Materials Processing and Mold (Zhengzhou University), Ministry of Education; National Engineering Research Center for Advanced Polymer Processing Technology, Zhengzhou University, Zhengzhou 450002, P. R. China

[2]Department of Chemistry, College of Science, Taif University, P.O.Box 11099 Taif 21944, Saudi Arabia

[3]Mechanical and Construction Engineering, Faculty of Engineering and Environment, Northumbria University, Newcastle upon Tyne, NE1 8ST, UK

[4]Integrated Composites Laboratory (ICL), Department of Chemical & Biomolecular Engineering, University of Tennessee, Knoxville, TN 37996, USA

[5]Advanced Materials Division, Engineered Multifunctional Composites (EMC) Nanotech LLC, Knoxville, TN 37934, USA

[6] College od Materials Science and Engineering, Taiyuan University of Science and Technology, Taiyuan 030024, P. R. China

[7]Department of Mechanical & Manufacturing Engineering, Manipal Institute of Technology, Manipal Academy of Higher Education, Manipal-576104, Karnataka, India

[8]Department of Chemistry, Turabah University College, Taif University, P.O.Box 11099, Taif 21944, Saudi Arabia

[9] Department of Chemistry, Al-Azhar University, Nasr City 11884, Cairo, Egypt

*Corresponding authors. E-mail: fmsu@zzu.edu.cn (F. Su); ben.xu@northumbria.ac.uk (B. Xu); zguo10@utk.edu or nanomaterials2000@gmail.com (Z. Guo)

Supplementary Figures

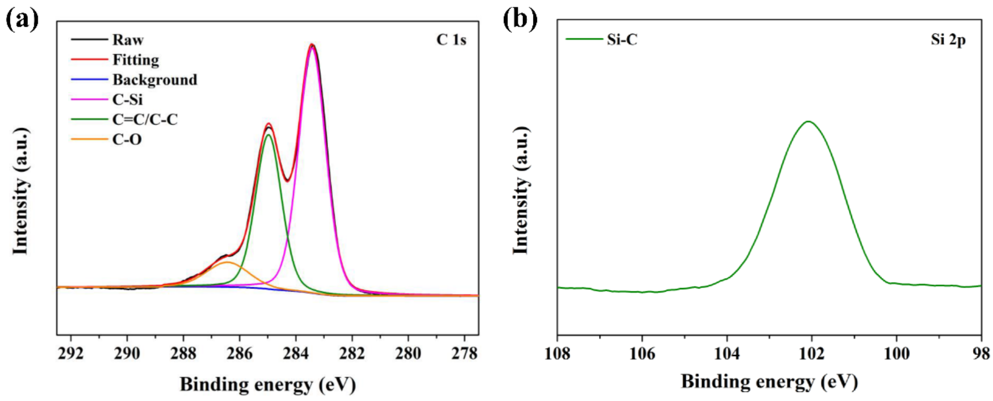

Fig. S1 High-resolution XPS analysis of **a** C 1s and **b** Si 2p of commercial SiC NWs

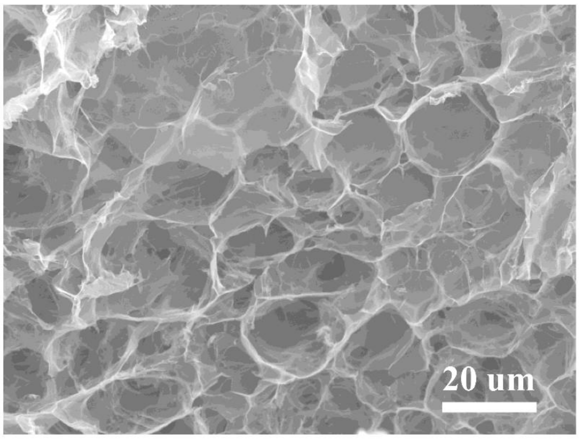

Fig. S2 SEM image of CA

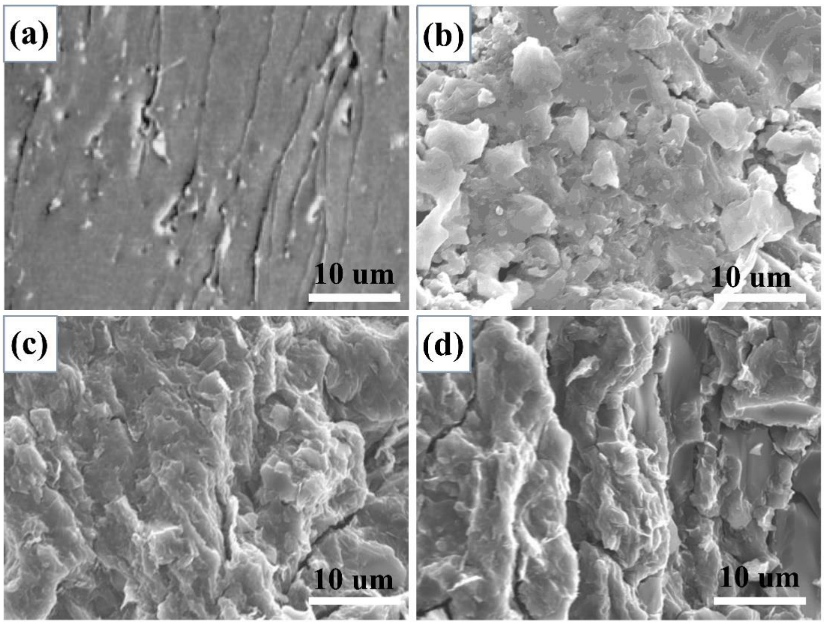

Fig. S3 SEM images of **a** CA/0.10m-SiC/EP, **b** CA/1.8m-BN/EP, **c** CA/0.10m-SiC/1.8m-BN/EP_{blend} and **d** CA/0.10m-SiC/1.8m-BN/EP

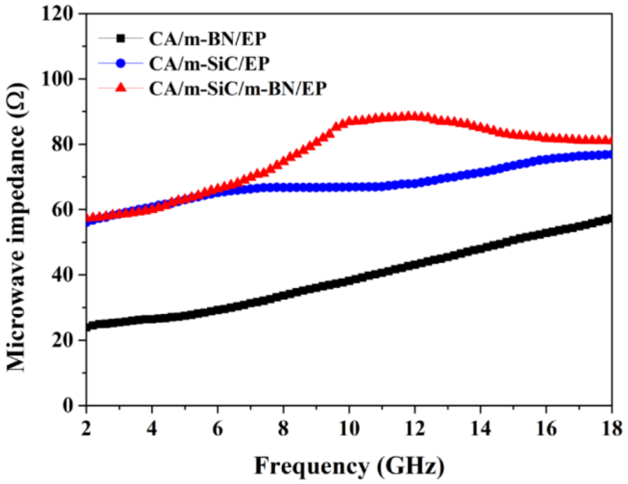

Fig. S4 The microwave impedance of CA/m-SiC/EP, CA/m-BN/EP and CA/m-SiC/m-BN/EP

Calculation process of filler content

Since the sample presents a regular cylinder, the density is measured according to the following Equation (S1):

$$\sigma = \frac{m}{V} \tag{S1}$$

where σ, m and V are the density, mass and volume of the different samples, respectively.

The filler content is obtained with the help of a thermal gravimetric analyzer. Since BN and SiC are both thermally stable materials with high temperature resistance, ignoring the small effects of surface adsorbed water and modified groups, the total filler loading is referred to Equation (S2):

$$\vartheta = \frac{m\prime}{m_0} \times 100\% \tag{S2}$$

where ϑ is the weight fraction of total filler, m_0 is the mass of the sample before testing, m' is the mass of the sample after the thermal analysis test at air atmosphere in 600 °C. So, the m-SiC NWs loading is obtained by Equation (S3):

$$\epsilon = \vartheta \times \frac{x}{x+y} \times 100\% \tag{S3}$$

And, the m-BN loading is obtained by Equation (S4):

$$\varphi = \vartheta \times \frac{y}{x+y} \times 100\% \tag{S4}$$

where ϵ and x are the mass content of m-SiC NWs and the mass added during sample synthesis, respectively; φ and y are the mass content of m-BN and the mass added during sample synthesis, respectively.

Table S1 The density of CA/m-SiC/m-BN/EP composites with different m-SiC NWs contents at the m-BN mass of 1.8 g, and the corresponding filler loadings

Samples	Density (g cm^{-3})	m-SiC NWs (wt%)	m-BN (wt%)	Filler loading (wt%)
CA/1.8m-BN/EP	1.145	0.00	14.37	14.37
CA/0.03m-SiC/1.8m-BN/EP	1.116	0.24	14.42	14.66
CA/0.06m-SiC/1.8m-BN/EP	1.083	0.50	14.89	15.39
CA/0.10m-SiC/1.8m-BN/EP	1.032	0.88	15.81	16.69
CA/0.13m-SiC/1.8m-BN/EP	0.958	1.25	17.25	18.50

Table S2 The density of CA/m-SiC/m-BN/EP composites with different m-BN contents at the m-SiC NWs mass of 0.10 g, and the corresponding filler loadings

Samples	Density (g cm^{-3})	m-SiC NWs (wt%)	m-BN (wt%)	Filler loading (wt%)
CA/0.10m-SiC/EP	0.732	0.74	0.00	0.74
CA/0.10m-SiC/0.5m-BN/EP	0.841	0.81	4.03	4.84
CA/0.10m-SiC/1.0m-BN/EP	0.927	0.82	8.20	9.02
CA/0.10m-SiC/1.5m-BN/EP	0.989	0.87	13.04	13.91
CA/0.10m-SiC/1.8m-BN/EP	1.032	0.88	15.81	16.69

Table S3 A comparison of the *TC* of the bulk composites reported in some recent literatures

Composites	Filler content	*K* (W/mK)	Refs.
AlN/C/EP	12.4 wt%	0.49	2022 [S1]
BN/PVMQ	31 vol%	1.64	2022 [S2]
CNTs/PP@Gr	CNTs 2wt%<br>Gr 13.01wt%	0.82	2022 [S3]
ND@SCNWs/PDMS	25 wt%	0.57	2022 [S4]
PEEK/MWCNT/GNS	MWCNT 5wt%<br>GNS 20wt%	2.64	2022 [S5]
BN-rGO/NR	12 wt%	1.04	2022 [S6]
BN/MXene/PVA	44 wt%	1.51	2021 [S7]
QAC/BN/PP	10 wt%	0.88	2021 [S8]
Al_2O_3/EP	17.7 wt%	0.72	2021 [S9]
BN@AgNWs/PDMS	20 vol%	0.91	2021 [S10]
EVA@BN/EP	16.8 vol%	1.85	2021 [S11]
BN/CNTs/TPU	65 wt%	1.35	2020 [S12]
BN@CNTs/PBz	25 wt%	0.79	2020 [S13]
BNNS/PDMS	35 wt%	1.16	2020 [S14]
BN/Fe_3O_4/EP	20 wt%	1.07	2020 [S15]
CA/m-SiC/m-BN/EP	16.69 wt%	2.21	This work

Notes: AlN: Aluminum Nitride, PVMQ: phenyl silicone rubber, CNTs: carbon nanotubes, PP: polypropylene, Gr: Graphite, ND: nano-crystalline diamonds, SCNWs: SiC nanowires, PDMS: polydimethylsiloxane, PEEK: poly (ether ether ketone), MWCNT: multi-walled carbon nanotube, GNS: graphite nanosheet, rGO: reduced graphene oxide, NR: natural rubber, PVA: Poly(Vinyl Alcohol), QAC: Quaternary ammonium salt, EVA: poly (ethylene-co-vinylacetate), TPU: polyurethane, PBz: polybenzoxazine.

Supplementary References

[S1] Z. Chen, C. Zhao, W. Meng, N. Sheng, C. Zhu et al., Anisotropically enhancing thermal conductivity of epoxy composite with a low filler load by an AlN/C fiber skeleton. Ceram. Int., (2022). https://doi.org/10.1016/j.ceramint.2022.03.030

[S2] M. Sheng, R. Yang, H. Gong, Y. Zhang, X. Lin et al., Enhanced thermal conductivity and stability of boron nitride/phenyl silicone rubber composites via surface modification and grain alignment. J. Mater. Sci. **57**(10), 5805-5824 (2022). https://doi.org/10.1007/s10853-021-06860-8

[S3] S. Hu, B. Xu, Y. Zhao, X. Fu, Q. Liu et al., Preparation of CNTs/PP@Gr composites with a segregated structure and enhanced electrical and thermal conductive properties by the pickering emulsion method. Compos. Sci. Technol. **222**, 109374 (2022). https://doi.org/10.1016/j.compscitech.2022.109374

[S4] Y. Qin, B. Wang, X. Hou, L. Li, C. Guan et al., Constructing tanghulu-like diamond@silicon carbide nanowires for enhanced thermal conductivity of polymer composite. Compos. Commun. **29**, 101008 (2022). https://doi.org/10.1016/j.coco.2021.101008

[S5] Z. Jiang, X. Liu, Q. Xu, C. Zhou, Y. Shang et al., Thermal conductive segregated multi-scale network constructed by ball-milling and in-situ polymerization in PEEK/MWCNT/graphite composite. Compos. Commun. **29**, 101035 (2022). https://doi.org/10.1016/j.coco.2021.101035

[S6] D. An, Z. Li, H. Chen, C. Liang, Z. Sun et al., Modulation of covalent bonded boron nitride/graphene and three-dimensional networks to achieve highly thermal conductivity for polymer-based thermal interfacial materials. Compos. Part A **156**, 106890 (2022). https://doi.org/10.1016/j.compositesa.2022.106890

[S7] S. Lee, J. Kim, Incorporating mxene into boron nitride/poly(vinyl alcohol) composite films to enhance thermal and mechanical properties. Polymers **13**(3), 379 (2021). https://doi.org/10.3390/polym13030379

[S8] S.W. Xiong, P.G. Fu, Q. Zou, L.Y. Chen, M.Y. Jiang et al., Heat conduction and antibacterial hexagonal boron nitride/polypropylene nanocomposite fibrous membranes for face masks with long-time wearing performance. ACS Appl. Mater. Interfaces **13**(1), 196-206 (2021). https://doi.org/10.1021/acsami.0c17800

[S9] C. Zhu, Z. Chen, R. Zhu, N. Sheng, Z. Rao, Vertically aligned Al_2O_3 fiber framework leading to anisotropically enhanced thermal conductivity of epoxy composites. Adv. Eng. Mater. **23**(9), 2100327 (2021). https://doi.org/10.1002/adem.202100327

[S10] Z. Huang, W. Wu, D. Drummer, C. Liu, Y. Wang et al., Enhanced the thermal conductivity of polydimethylsiloxane via a three-dimensional hybrid boron nitride@silver nanowires thermal network filler. Polymers **13**(2), 248 (2021). https://doi.org/10.3390/polym13020248

[S11] Y. Cui, D. Bao, F. Xu, Y. Gao, X. Zhang et al., Fabrication of eva connected 3D BN network for enhancing the thermal conductivity of epoxy composites. Compos. Part B **224**, 109203 (2021). https://doi.org/10.1016/j.compositesb.2021.109203

[S12] Y. Ruan, N. Li, C. Liu, L. Chen, S. Zhang et al., Increasing heat transfer performance of thermoplastic polyurethane by constructing thermal conduction channels of ultra-thin boron nitride nanosheets and carbon nanotubes. New J. Chem. **44**(43), 18823-18830 (2020). https://doi.org/10.1039/d0nj04215c

[S13] Y. Wang, W. Wu, D. Drummer, C. Liu, F. Tomiak et al., Achieving a 3D thermally conductive while electrically insulating network in polybenzoxazine with a novel hybrid filler composed of boron nitride and carbon nanotubes. Polymers **12**(10), 2331 (2020). https://doi.org/10.3390/polym12102331

[S14] M. Li, M. Wang, X. Hou, Z. Zhan, H. Wang et al., Highly thermal conductive and electrical insulating polymer composites with boron nitride. Compos. Part B **184**, 107746 (2020). https://doi.org/10.1016/j.compositesb.2020.107746

[S15] Y. Kim, J. Kim, Fabrication of Fe_3O_4 coated boron nitride nanoplatelets by liquid-phase exfoliation for thermally enhanced epoxy composites via magnetic alignment. Compos. Sci. Technol. **188**, 107961 (2020). https://doi.org/10.1016/j.compscitech.2019.107961
